# Descriptive analysis of pharmacy services provided after community pharmacy screening

**DOI:** 10.1007/s11096-018-0742-5

**Published:** 2018-11-26

**Authors:** Karla Lancaster, Lehana Thabane, Jean-Eric Tarride, Gina Agarwal, Jeff S. Healey, Roopinder Sandhu, Lisa Dolovich

**Affiliations:** 1 McMaster University, 1280 Main St. W., Hamilton, ON L8S 4L8 Canada; 2 Population Health Research Institute, 237 Barton St. E., Hamilton, ON L8L 2X2 Canada; 3 University of Alberta, 116 St. and 85th Ave., Edmonton, AB T6G 2R3 Canada; 4 Leslie Dan Faculty of Pharmacy, University of Toronto, 144 College St., Room 607, Toronto, ON M5S 3M2 Canada

**Keywords:** Canada, Chronic disease, Mass screening, Medication, Pharmaceutical services, Pharmacies, Review

## Abstract

*Background* Community pharmacies are promising locations for opportunistic screening due to pharmacist accessibility and ability to perform various health and medication management services. Little is known as to the provision of pharmacy services following screening initiatives. *Objective* To describe provision of pharmacy services for participants following a community pharmacy stroke screening initiative. *Setting* The Program for the Identification of “Actionable Atrial” Fibrillation Pharmacy initiative took place in 30 pharmacies in Alberta and Ontario, Canada. 1149 participants ≥ 65 were screened for atrial fibrillation, type 2 diabetes, and hypertension. *Method* Retrospective, secondary analysis of data using participant case-report forms, pharmacy data, and pharmacy claims to describe pharmacy services received by participants post-screening. *Main Outcome Measure* Number and types of remunerated pharmacy services received by participants post-screening. *Results* A total of 535/1149 (46.6%) participants screened at their regular pharmacy were included in this analysis. Of these, 165 (30.8%) participants received 229 pharmacy services within 3 months post-screening, including 146 medication reviews, 57 influenza vaccinations, and 21 pharmaceutical opinions. A median (interquartile range, IQR) of 6 (2–11) pharmacy services were delivered, and median (IQR) reimbursement was $187.50 ($67.50–$342.50). *Conclusions* Approximately one-third of participants received a pharmacy service within 3 months post-screening. Relatively large numbers of annual and follow-up medication reviews were delivered despite low eligibility for annual-only reviews and despite many missed opportunities for pharmacy service provision in at-risk patients. In-pharmacy screening may facilitate provision of some services, namely medication reviews, by providing opportunities to identify patients at-risk.

**Electronic supplementary material:**

The online version of this article (10.1007/s11096-018-0742-5) contains supplementary material, which is available to authorized users.

## Impacts on practice

- In-pharmacy screening initiatives represent an opportunity for patient follow-up, which may include provision of remunerated pharmacy services.
- The most frequently provided services post screening in Canada are medication reviews, influenza vaccinations and pharmaceutical opinions.
- Despite low eligibility for annual reviews, high numbers of both annual and follow-up medication reviews are provided after in-pharmacy screening initiative.
- Participants in a pharmacy screening program may be willing to attend CVD or diabetes screening sessions in locations other than their home pharmacy.

## Introduction

Within Canada and around the world, pharmacist practice is shifting away from dispensing activities and towards a patient-centred model of care [1–5]. Community pharmacists are increasingly focusing on medication management services, including medication reviews, prescription adaptation and extension, smoking cessation consultations, and independent prescribing [6–9]. These services are useful for identifying medication-related problems, and for ongoing patient monitoring and follow-up, especially where chronic disease is present.

In addition to medication management services, other activities such as community pharmacy-based opportunistic screening for chronic and cardiovascular disease (CVD) risk factors, are gaining popularity. Opportunistic screening, defined as screening that is “carried out at a time when people are seen, by health care professionals, for a reason other than the disorder in question”, is cost-effective, can help detect and prevent chronic disease, and can be effective at reducing morbidity [10–22]. Screening for CVD, diabetes and stroke risk factors in community pharmacies offers the possibility of linking people with newly-identified potential health risks to an on-site pharmacist. Pharmacists can provide guidance on screening results, help navigate the healthcare system (including encouraging patients to speak with their family physician), and provide recommendations and services to improve medication management. Community pharmacists thus have an emerging role in CVD, diabetes and stroke prevention and management, augmented by their accessibility and role as experts in the safe and effective use of medications [9]. However, it is imperative that follow-up care is appropriate and high-quality [16, 22, 23].

Few studies have investigated how remunerated pharmacy services are provided following community pharmacy screening initiatives for CVD, stroke prevention or diabetes. Many screening initiatives defer patient follow-up to physicians, or have pharmacists provide patient education post-screening; very few discuss use of formal, remunerated services such as medication reviews. Furthermore, most pharmacy screening initiatives include patient counselling or education, rather than exploring their use as part of usual practice *after* the activity is complete. This study investigated the use of remunerated pharmacy services following an in-pharmacy initiative, the Program for Identification of “Actionable” Atrial Fibrillation pharmacy study (PIAAF), that provided opportunistic screening and risk assessment for atrial fibrillation (AF), hypertension and type 2 diabetes to community-dwelling individuals. Combining AF screening for stroke prevention with assessment for other health risks provided an opportunity to identify those who could benefit the most from focused attention, including those with more than one health issue.

## Aim of study

The objective of this secondary analysis, the PIAAF-professional pharmacy service analysis (PIAAF-PPS), was to describe the extent (number, type, remuneration) of remunerated service delivery post-PIAAF screening in two Canadian provinces, Ontario and Alberta. These jurisdictions are reported separately due to differences in available remunerated services between provinces (Tables 2 and 3). It was hypothesized that in both jurisdictions, the PIAAF initiative would encourage more services to be provided to study participants. This study sought to provide a better real-world understanding of gaps and continuity of care between screening initiatives and pharmacist services. Most research deals with these two activities separately or as part of very structured research protocols (i.e. randomized controlled trials).

## Ethics approval

This study was approved by the Hamilton Integrated Research Ethics Board. A formal data sharing agreement between McMaster University and Rexall pharmacies was in place to allow sharing of pharmacy records with the study team (with participant consent). Informed consent was obtained from all individual participants included in the study.

## Methods

### PIAAF pharmacy

The PIAAF study was an organized, community pharmacy-based initiative to screen community-dwelling elders for AF and hypertension, and assess risk for type 2 diabetes [23, 24]. Approximately 1175 seniors (> 65 years) from Hamilton, Ontario, and Edmonton, Alberta attended screening/assessment sessions in 30 Rexall chain pharmacies between October 2014 and April 2015. AF was screened using a single-lead, handheld electrocardiogram (ECG), diabetes risk was assessed using the CANRISK tool [25], and blood pressure (BP) was measured using the PharmaSmart in-pharmacy kiosk [26]. Participants screening positive for AF were recommended to receive a 12-lead ECG, either through their family physician or an AF clinic. Results for participants at risk for any factor were sent to their family physician. Community pharmacists were not formally referred to counsel individuals identified at-risk, however, participants were encouraged to speak to the pharmacist about their results. The intention was to mimic usual practice in pharmacies where screening opportunities are provided (such as through BP kiosks) without a mandatory pharmacist appointment.

### Participants and pharmacies

PIAAF participants who reported that the pharmacy where they were screened/assessed was also their primary or “home” pharmacy were included in this analysis. Participants with a different home pharmacy were also included *if* a partial pharmacy profile was located, or if they received a pharmacy service at the pharmacy where they were screened. Pharmacies were included if data extraction was possible, and if > 1 eligible participant received screening/assessment at that location.

### Data sources

Data were collected from two sources: case report forms (CRFs) for each participant, collected at the time of assessment; and pharmacy profile and administrative billing data.

Data extracted from CRFs included: (1) name and date of birth; (2) pharmacy where screening/assessment was performed, and whether this was their home pharmacy; (3) date of screening/assessment; (4) self-reported medication use; (5) self-reported medical conditions; (6) BP measurements; (7) AF screening results; (8) CANRISK assessment results in those < 74 years without known diabetes; and (9) smoking status.

Where participant pharmacy profiles (either full or partial) were located at the screening/assessment pharmacy, the following data were extracted: (1) audit histories for all product or drug information numbers (PINS/DINS) billed from October 1, 2014 to October 31, 2015 including fill date, quantity authorized and dispensed, and status (e.g. completed, cancelled); (2) any chronic conditions/diagnoses listed. This was used to calculate number of medications taken, and identify new or potentially inappropriate medications dispensed (assessed using the Beers List criteria) [27]. Of 1149 enrolled PIAAF study participants, 614 (53.4%) were excluded from the PIAAF-PPS analysis because screening sessions were not held at their home pharmacy (n = 575), or because profiles were inactivated (e.g. following death or admission into long-term care) or otherwise unable to be located (e.g. due to incorrectly transcribed data on CRFs) (n = 39). Partial profiles for 45 participants were found. Therefore, 535 (46.6%) PIAAF participants from 26 pharmacies were included. Demographic information, including self-reported medication use, was collected for all 535 participants. Pharmacy claims data was considered the ‘gold standard’; however, some claims data could not be retrieved, or was incomplete. In these cases, self-reported data from CRFs was used to impute number of medications (n = 44).

### Data analysis

Descriptive statistics were performed for the mean number of pharmacy services provided within 3 months of screening, per participant; median number of services provided, per region and pharmacy; median dollar amount reimbursed for services, per region and pharmacy; and counts of each type of pharmacy service provided on the day of assessment, within the first week, and within 3 months of assessment, per region and pharmacy. Both provinces were compared to investigate whether the difference in available remunerated services impacted pharmacy service delivery. Only the dollar amounts reimbursed to pharmacies for remunerated services were reported, as pharmacies did not incur any cost of intervention. An economic analysis of the PIAAF Pharmacy screening initiative (i.e. not taking into account pharmacy services) has been reported elsewhere [24]. Chi square tests were performed to investigate whether there were significant differences in patient demographics and medication use between jurisdictions. Analyses were performed using SPSS v23.

## Results

**Fig. 1:**
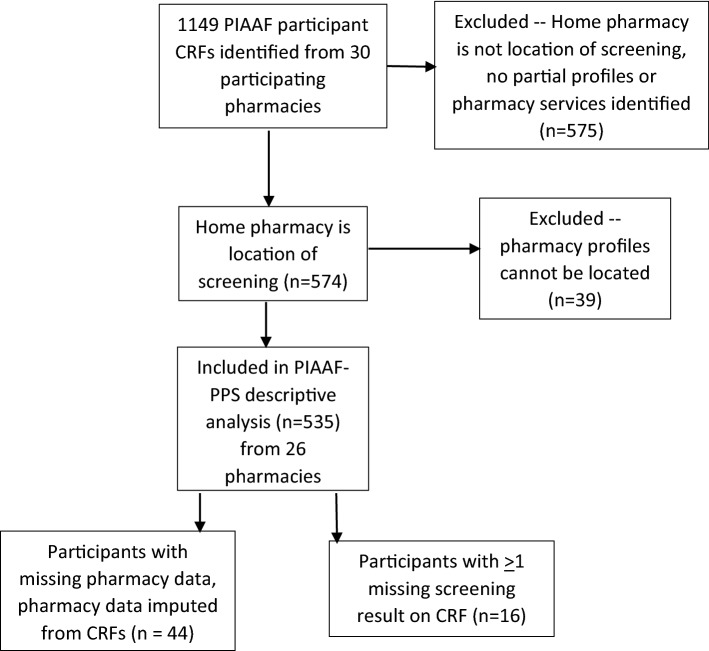
Flow chart of PIAAF pharmacy participants through the PIAAF-PPS descriptive analysis

Of the 535 participants included in this analysis, 404 (76%) were from Ontario, and 131 (24%) were from Alberta. Figure 1 demonstrates the flow of participants from the PIAAF study through the PIAAF-PPS analysis.

Of 30 participating pharmacies, 26 (87%) were included. One pharmacy closed following PIAAF, and one did not utilize Rexall’s proprietary software system (thus, data could not be extracted). The remaining two did not enroll any regular customers; no pharmacy services were linked to participants assessed at those stores.

### Baseline characteristics

Baseline characteristics for PIAAF-PPS participants are reported in Table 1. The mean age (SD) was 75.4 (6.8), and 56% were female. History of hypertension was reported in 299 (56%) participants, known diabetes was reported in 120 (22.4%), and 8 (1.5%) reported pre-diagnosed AF. Only 32 (6.0%) were smokers.

**Table 1:** Baseline characteristics of participants included in PIAAF-PPS analysis

Participant characteristics	n (%) n = 535
Age: Mean (SD)	75.4 (6.8)
Female participants	301 (56.3)
Smoker	32 (6.0)
Known atrial fibrillation	8 (1.5)
History of hypertension	299 (55.9)
High blood pressure at time of screening	160 (29.9)
Known diabetes mellitus	120 (22.4)
History of heart failure	19 (3.6)
History of vascular artery disease	64 (12.0)
History of stroke or TIA	48 (9.0)
Participants with polypharmacy (≥ 4 medications)^a^	237 (48.3)
Mean (SD) number of medications	4.4 (3.3)
Participants who received any pharmacy service within 1 year prior to screening	351 (66.6)
Participants eligible for annual-only medication review on day of screening or within 3 months post-screening	194 (36.3)
Participants with ≥ 1 high risk medication^a^	192 (39.1)

^a^N = 491 participants with available medication data

### Pharmacy services

165 participants received 229 pharmacy services within 3 months post-screening. These included: 145 medication reviews [71 (49%) annual-only, 74 (51%) follow-ups], 57 influenza vaccinations, 21 pharmaceutical options (Ontario), 4 assessments for prescription renewals (Alberta), 1 assessment for prescription adaptation (Alberta), and 1 smoking cessation consultation (Ontario). Participants received an average (SD) of 0.43 (0.76) pharmacy services, with an average (SD) of 1.43 (0.73) in those receiving > 1 service. There was a large variation in the number of pharmacy services provided per pharmacy, ranging from 0 to 45 with a median (IQR) of 6 (2–11). 351 (67%) participants had received any remunerated service in the year prior to screening/assessment, and 194 (36%) participants were (or became) eligible for an annual-only review during the post-screening initiative period. Of these 194 eligible participants, 71 (37%) received an annual-only medication review during this timeframe. In total, 66 services were provided on the date of screening/assessment, of which 35 (53%) were medication reviews.

The median dollar amount (IQR) reimbursed per pharmacy for remunerated services was $187.50 ($67.50–$342.50).^1^ In total, participating pharmacies were reimbursed $7,877.50 for pharmacy services billed for participants within 3 months of screening/assessment. Stores in Ontario tended to provide more services, especially on the day of screening. Stores in Alberta provided a larger proportion of medication reviews compared to Ontario.

#### Ontario

During the 3 month post-screening initiative period, 127 (31%) Ontario participants received 167 pharmacy services: 98 (59%) medication reviews, 47 (28%) influenza vaccinations, 21 (13%) pharmaceutical opinions, and 1 (0.6%) smoking cessation consultation (Table 2). 128 (32%) participants were eligible for annual-only medication reviews: 57 (58%) of the identified medication reviews received were annual-only and 41 (42%) were follow-ups. The number of services provided per pharmacy ranged from 1 to 45, with a median (IQR) of 8 (4–14). 51 participants (40%) received 54 services (32%) on the day of the screening, 14 participants (11%) received 14 services (8%) in the first week post-screening, and 81 (64%) participants received 99 services (59%) during the remaining 3-month period. The total dollar value reimbursed per pharmacy for remunerated services ranged from $60.00 to $1780.00, with a median (IQR) per pharmacy of $263.75 ($120.00–$347.50).

**Table 2:** Pharmacy services billed for PIAAF pharmacy participants (n = 127) in Ontario

Pharmacy service	PIN billed	Services billed day of screening	Services billed within 1 week of screening	Services billed within 3 months of screening	Total
MedsCheck					
MedsCheck Annual	93899979	13	2	27	42
MedsCheck Annual Follow-up: pharmacist documented decision	93899982	6	2	16	24
MedsCheck Diabetes: follow-up	93899989	8	1	7	16
MedsCheck Diabetes	93899988	3	0	11	14
MedsCheck Hospital Discharge: follow up	93899981	0	0	1	1
MedsCheck At Home	93899987	0	0	1	1
Influenza vaccination					
Influenza vaccine: FLUVIRAL	02015986	16	2	10	28
Influenza vaccine: AGRIFLU	02346850	7	5	7	19
Pharmaceutical Opinions					
Pharmaceutical Opinion program (POP): change to prescription	93899993	1	2	11	14
POP: No change to prescription	93899992	0	0	6	6
POP: prescription not filled	93899991	0	0	1	1
Other services					
Pharmacy smoking cessation program: initial	93899941	0	0	1	1
Total	–	54	14	99	167
Number of participants who received ≥ 1 pharmacy service	–	51	14	81	146^a^

^a^127 individuals in ON received pharmacy services, however, 19 received > 1 service in two different time periods, and therefore these people are counted twice in this total

#### Alberta

During the 3 month post-screening initiative period, 38 (29.0%) Alberta participants received 62 pharmacy services: 47 (75.8%) medication reviews, 10 (16.1%) influenza vaccinations, 4 (6.5%) assessments for prescription renewal, and 1 (1.6%) assessment for prescription adaptation. 66 (50%) participants were eligible for an annual-only medication review: 14 (30%) of the identified medication reviews were annual-only, and 33 (70%) were follow-ups (Table 3). The number of services provided per pharmacy ranged from 0 to 20, with a median (IQR) of 4.5 (1–6). 19.4% (12/62) of services occurred on the same day as screening. 11 participants (29%) received 12 services (19%) on the day of screening/assessment, 4 participants (11%) received 4 services (6%) in the first week post-screening, and 30 (79%) participants received 46 services (74%) during the remaining 3-month period. The dollar amount reimbursed per pharmacy for remunerated services ranged from $0.00 to $970.00, with a median (IQR) of $132.50 ($22.50–$232.50).

**Table 3:** Breakdown of pharmacy services billed for PIAAF pharmacy participants in Alberta

Pharmacy service	PIN billed	Services billed day of screening	Services billed within 1 week of screening	Services billed within 3 months of screening	Total
Comprehensive Annual Care Plans (CACPs)					
Follow-up CACP	00000071115	2	1	17	20
	00000081115	0	0	8	8
CACP	00000071114	0	2	6	8
	00000081114	1	0	3	4
Standard medication management assessments (SMMAs)					
SMMA follow-up: chronic disease	00000071113	2	0	2	4
	00000081113	0	0	1	1
SMMA: chronic disease	00000071112	0	1	1	2
	00000081112	0	0	0	0
Influenza vaccination					
Immunization: routine recommended immunization	05666650	7	0	3	10
Prescription adaptation and prescriptive authority					
Assessment for prescription renewal	00000071111	0	0	4	4
	00000081111	0	0	0	0
Assessment for adaptation of a prescription	00000071111	0	0	1	1
	00000081111	0	0	0	0
Total	–	12	4	46	62
Number of participants who received ≥ 1 pharmacy service	–	11	4	30	45^a^

^a^38 individuals in AB received pharmacy services, however, 7 received > 1 service in two different time periods and therefore these people are counted twice in this total

### Screening results

Results for > 1 screening test were missing from 16 (3%) CRFs. 15 (2.8%) participants screened positive for AF. Of these, Sandhu et al. [23] reported that 9 spoke to the pharmacist after screening, and 9 had OAC subsequently prescribed by a specialist or family physician. 529 (99%) participants screened had a CHA2DS2-VASc score of > 1, indicating a low-to-moderate risk of stroke [28]. 160 (30%) participants had raised BP at the time of screening, including 54 (10.3%) participants with known diabetes. Mean (SD) BP was 139.7/75.6 mmHg (21.2/12.1 mmHg). 203 participants < 74 years were risk-assessed for diabetes using the CANRISK questionnaire: 99 (48.8%) were at high risk, and 88 (43.3%) were at intermediate risk. Assessment results are presented in Table 4.

**Table 4:** Screening results from participants included in PIAAF-PPS analysis

Screening results	N (%)
Completed CANRISK screening for diabetes mellitus (n = 203)	
Low risk for diabetes	16 (7.9)
Intermediate risk for diabetes	88 (43.3)
High risk for diabetes	99 (48.8)
Screened for atrial fibrillation with single-lead ECG (n = 535)	
AF screened positive	15 (2.8)
CHA2DS2—vasc score ≥ 1	529 (98.9)
CHA2DS2—vasc score < 1	6 (1.1)
Screened for hypertension (n = 526)	
Hypertension, no diabetes	106 (20.2)
Hypertension, diabetes	54 (10.3)
No hypertension, no diabetes	299 (56.8)
No hypertension, diabetes	65 (12.4)
No hypertension, unsure of diabetic status^a^	2 (0.4%)
Mean systolic blood pressure (SD)	139.7 (21.2)
Mean diastolic blood pressure (SD)	75.6 (12.1)
Mean heart rate (SD)	71.3 (12.4)

^a^Based on self-report in CRFs

### Medication use

Participants took a mean (SD) of 4.4 (3.3) medications, ranging from 0 to 19. Polypharmacy was identified in 237 (48%) participants (defined here as use of > 4 concurrent medications). The most commonly used medications were statins (50%), and low-dose ASA (54%). Medication use per jurisdiction (self-reported and from pharmacy claims data) is reported in supplemental Table 1.

## Discussion

Approximately one-third of PIAAF participants received at least one remunerated service within 3 months post-screening initiative. Most billed pharmacy services were medication reviews, accounting for approximately two-thirds of identified services. Other services (e.g. prescription adaptation, pharmaceutical opinions, and smoking cessation consultations) were far less frequent. Participant screening/assessment represented an opportunity for subsequent pharmacist intervention; this study shows that provision of pharmacy services to PIAAF participants generated just under $7, 880.00 of revenue for participating pharmacies. This number could have been higher had all PIAAF participants at risk for stroke, diabetes or CVD received a medication review; therefore, not all opportunities for patient follow-up were capitalized upon. Had *all* PIAAF participants (not just those included in this analysis) screening positive for AF, at high- or intermediate-risk for diabetes, with history of hypertension, or with high BP at time of screening received a medication review (annual or follow-up, based on eligibility), the total dollar amount reimbursed across all participating pharmacies would have been approximately $33,000.00 (calculated using pricing for non-APA SMMAs in Alberta). Nevertheless, many PIAAF participants received a medication review on the same day of screening/assessment, demonstrating that in-pharmacy screening/assessment and provision of pharmacy services can be successfully combined, especially when screening sessions are planned ahead of time, as was the case with the PIAAF program. Although influenza vaccinations were the second-most common service provided, uptake remained low. This may be due to the fact that approximately half of the screening sessions were not held during the annual flu-shot season (roughly October–December). Previous research has also shown that older adults are more likely to receive flu shots at their physician’s office [29].

Many PIAAF participants were found to be at risk for stroke, diabetes, hypertension, or a combination of these. The number of participants screening positive for AF was comparable to a population prevalence of approximately 3–5% in those > 65 (US data) [30]. Almost all participants were found to have moderate risk of stroke or higher [23]. The majority assessed with CANRISK were found to be at high or intermediate risk of diabetes. This was on top of those with known diabetes, a rate which was consistent with a reported prevalence of approximately 16–25% in Canadians aged > 65 [31]. The prevalence of hypertension was also consistent with Canadian averages (roughly 60%) [32]; just under a third of participants had raised BP at the time of screening. Some of these participants may have benefitted from additional BP control. Despite a comparatively low average number of medications [6, 33], polypharmacy was present in almost half of participants. Altogether, this represents a large proportion of participants that may have benefited from pharmacist-delivered monitoring and follow-up services like medication reviews (including MedsCheck diabetes and diabetes-focused SMMAs), pharmaceutical opinions (in Ontario) or prescriptive authority (in Alberta).

Some pharmacies were reimbursed more for providing less, but more intensive, time-consuming services (e.g. medication reviews) than pharmacies that performed more, but quicker, less complex services (e.g. influenza vaccinations). This was especially true in Alberta, where the vast majority of provided services were medication reviews. Considering that approximately one-third of participants were eligible for an annual medication review during the post-screening period, the number of medication reviews provided was high; in fact, just over half of identified reviews were follow-ups. Pharmacists likely feel there is a trade-off between time spent performing pharmacy services and reimbursement [34]. Low smoking cessation consultation rates in both provinces were likely related to the very low smoking rate observed in this cohort compared to the Canadian rate of 18.1% [35], but may also be due to low implementation of these services in community pharmacies, as has been seen in Ontario [36].

This study had several limitations. Because participant pharmacy data could only be extracted for participants who attended screening sessions at their home pharmacy, over half of PIAAF participants were excluded. This reduced the available sample size and the number of identified pharmacy services. The fact that over half of PIAAF participants were not screened in their home pharmacy may suggest that many people were interested enough in their screening/assessment results that they were willing to participate outside of their regular health care setting. Pharmacies in Ontario recruited more regular pharmacy patrons than in Alberta and provided more services; two Alberta sites provided no pharmacy services for PIAAF participants, and two provided one service each. This disparity demonstrates that while pharmacy service provision is possible within certain pharmacy workflows, it was not widespread or consistent even within one pharmacy organization. This study also does not consider services that were recommended but not accepted. There was no comparison group to allow comparisons between people who did and did not participate in the screening initiative. Strengths of this study include a large number of participating pharmacies and a relatively large number of eligible participants, even after exclusions were made. To our knowledge, this is the first Canadian study investigating use of pharmacy services in everyday practice following a three-pronged screening and assessment initiative. Screening initiatives such as PIAAF may have some potential to increase the provision of pharmacy services for those at risk, while also bolstering pharmacy revenue via remuneration for these services. Further research, including analyses of larger administrative datasets that can link routinely collected data from screening initiatives with reimbursed pharmacy services or pragmatic randomized controlled trials, would be necessary to substantiate these findings.

## Conclusions

Approximately one-third of participants attending a community pharmacy screening initiative for AF, hypertension and diabetes received at least one remunerated pharmacy service within 3 months. Medication reviews were the most frequently provided service, followed by influenza vaccinations, and pharmaceutical opinions. A relatively high number of eligible participants received an annual-only review, and a large number of follow-up reviews were also identified. With the exception of influenza vaccinations, most services were provided more than 1 week post-screening; however, many medication reviews were also performed on the day of screening. While there was considerable inter-pharmacy variation in the number of post-screening services provided, this study shows that pharmacies could potentially receive large amounts of reimbursement for providing remunerated services to participants screening at risk. However, many potentially useful services (e.g. prescribing interventions) were underutilized, indicating that a greater opportunity for the provision of services exists than was capitalized upon. Pharmacy service provision following in-pharmacy screening could potentially be augmented by direct pharmacist involvement in screening, e.g. by entering screening results into patient profiles. Overall, this study provides some evidence that community pharmacy screening sessions may help facilitate provision of remunerated services, especially medication reviews.

## Electronic supplementary material

Below is the link to the electronic supplementary material.

Supplementary material 1 (DOCX 14 kb)

## Footnotes

1 All dollar amounts are reported in CAD.
