# Supplementary material for: Descriptive analysis of pharmacy services provided after community pharmacy screening
Source: Int J Clin Pharm. 2018 Nov 26;40(6):1577–86. doi: 10.1007/s11096-018-0742-5 (PMC6280862; doi:10.1007/s11096-018-0742-5)
Supplement: Supplementary file 1 — Supplementary material 1 (DOCX 14 kb) [file 11096_2018_742_MOESM1_ESM.docx]

Supplemental Tables

**Table 1** Medication use in PIAAF-PPS cohort, self-reported and pharmacy claims, by region

| **Medication Class** | **All participants n (%)**  **N =535** | | **Edmonton Region n (%)**  **N =131** | | **Hamilton Region n (%)**  **N =404** | |
| --- | --- | --- | --- | --- | --- | --- |
|  | Self-Report | Pharmacy Claims | Self-Report | Pharmacy Claims | Self-Report | Pharmacy Claims |
| Low-dose ASA | 250 (46.7) | 245 (49.9) | 51 (38.9) | 52 (46.8) | 199 (49.3) | 193 (50.8) |
| Diuretics | 116 (21.7) | 135 (27.5) | 22 (16.8) | 30 (27.0) | 94 (23.3) | 105 (27.6) |
| Beta blocker | 109 (20.4) | 106 (21.6) | 16 (12.2) | 17 (15.3) | 93 (23.1) | 89 (23.4) |
| Calcium channel blocker | 106 (19.8) | 103 (21.0) | 22 (16.8) | 20 (18.0) | 84 (20.8) | 83 (21.8) |
| Angiotensin II receptor blocker | 109 (20.4) | 113 (23.0) | 32 (24.4) | 33 (29.7) | 77 (19.1) | 80 (21.1) |
| Insulin | 27 (5.0) | 28 (5.7) | 3 (2.3) | 4 (3.6) | 24 (5.9) | 24 (6.3) |
| Oral hypoglycemic | 86 (16.1) | 86 (17.5) | 24 (18.3) | 23 (20.7) | 62 (15.3) | 63 (16.6) |
| Statin | 264 (49.3) | 265 (54.0) | 49 (37.4) | 48 (43.2) | 215 (53.2) | 217 (57.1) |
| Alpha blocker | 15 (2.8) | 31 (6.3) | 2 (1.5) | 5 (4.5) | 13 (3.2) | 26 (6.8) |
| ACE inhibitor | 124 (23.2) | 135 (27.5) | 22 (16.8) | 21 (18.9) | 102 (25.2) | 114 (30.0) |
| Anti-arrhythmic | 1 (0.19) | 2 (0.41) | 0 (0) | 0 (0) | 1 (0.25) | 2 (0.53) |

**Table 2** Breakdown of numbers and dollar amounts reimbursed for remunerated pharmacy services billed for PIAAF-PPS cohort participants, per pharmacy

|  | **Services Billed Day of Screening** | **Services Billed within One Week of Screening** | **Services Billed within 3 months of Screening** | **Total** |
| --- | --- | --- | --- | --- |
| Median number of services billed per site (IQR),  all regions: | 1 (0-3) | 0 (0-1) | 4 (2-6) | 6 (2-11) |
| Hamilton region | 2.5 (0-7) | 0.5 (0-1) | 4.5 (3-7) | 8 (4-14) |
| Edmonton region | 1 (0-1.5) | 0 (0-0.5) | 3 (0.5-4.5) | 4.5 (1-6) |
| Median dollar amount reimbursed for services (IQR), all regions: | 20 (0-82.5) | 0 (0-25) | 128.75 (40-342.5) | 187.5 (60-342.5) |
| Hamilton region | 60 (0-181.25) | 3.75 (0-45) | 171.25 (77.5-260.0) | 263.75 (120-347.50) |
| Edmonton region | 20 (0-50) | 0 (0-10) | 72.5 (12.5-202.5) | 132.5 (22.5-232.5) |

S
